# Efficacy and safety of lurasidone in adolescents and young adults with schizophrenia: A pooled post hoc analysis of double-blind, placebo-controlled 6-week studies

**DOI:** 10.1192/j.eurpsy.2021.30

**Published:** 2021-05-10

**Authors:** Isabella Costamagna, Fabrizio Calisti, Agnese Cattaneo, Jay Hsu, Michael Tocco, Andrei Pikalov, Robert Goldman

**Affiliations:** 1 Angelini RR&D (Regulatory, Research, & Development), Angelini Pharma S.p.A., Viale Amelia, 70, 00181 Rome, Italy; 2 Sunovion Pharmaceuticals Inc., Fort Lee, New Jersey, USA; 3 Sunovion Pharmaceuticals Inc., Marlborough, Massachusetts, USA

**Keywords:** adolescent, atypical antipsychotic, lurasidone, schizophrenia, treatment-naïve

## Abstract

**Background:**

The aim of this pooled analysis was to evaluate the efficacy and safety of lurasidone in the treatment of an acute exacerbation of schizophrenia in adolescents and young adults.

**Methods:**

The six pooled studies in this analysis used similar study designs and outcome measures. Patients (aged 13–25 years) were randomized to 6 weeks of double-blind, placebo-controlled treatment with lurasidone in fixed doses of 40, 80, 120, or 160 mg. The primary efficacy endpoint was Week 6 change in the Positive and Negative Syndrome Scale (PANSS) total score; secondary efficacy endpoints included Week 6 change in the Clinical Global Impression–Severity scale.

**Results:**

The safety population consisted of 537 patients (mean age: 18.1 years); 82.6% of patients completed the studies. Treatment with lurasidone was significantly better than placebo at all doses (*p* < 0.001) for change in the PANSS total score at Week 6. Placebo-adjusted PANSS scores ranged from −9.4 to −16.1 (effect sizes: 0.53–0.90), with effect sizes increasing at higher doses. For lurasidone (combined doses), three adverse events occurred with a frequency of ≥5% (nausea: 13.5%; somnolence: 12.1%; akathisia: 10.1%). At last observation carried forward (LOCF)-endpoint weight gain of ≥7% was similar for lurasidone versus placebo (3.6 vs. 4.7%). Minimal median changes were observed at endpoint in cholesterol, −2.0 mg/dL; triglycerides, 0.0 mg/dL; and glucose, 0.0 mg/dL.

**Conclusions:**

In adolescents and young adults with schizophrenia, treatment with lurasidone in doses of 40–160 mg/d was a safe, well-tolerated, and effective treatment. Short-term treatment with lurasidone was associated with minimal effects on weight and metabolic parameters.

## Introduction

Schizophrenia is a debilitating brain disorder with a complex, polygenic architecture that interacts with environmental risk factors to confer disease susceptibility that commonly first manifests (at least in prodromal form) in adolescence [1,2]. Evidence suggests that earlier onset of schizophrenia (relative to latter onset) is associated with a poorer outcome characterized by more relapses and hospitalizations, more negative symptoms, and greater functional impairment [3]. Some research suggests that the negative effect of early onset is most evident in the first few years after initial diagnosis [4].

Atypical antipsychotics are recommended first-line treatments for schizophrenia; however, treatment of schizophrenia with these agents (and especially treatment of younger patients) has been associated with an increased risk of developing metabolic syndrome (e.g., abdominal adiposity, hyperlipidemia, and hyperglycemia). This has raised important safety concerns, especially given how frequently long-term treatment is required [5–11].

Lurasidone is an atypical antipsychotic agent with high binding affinity for D_2_, 5-HT_2A_, and 5-HT_7_ receptors (antagonist); moderate affinity for 5-HT_1A_ receptors (partial agonist); and no appreciable affinity for H_1_ and M_1_ receptors [12]. Lurasidone has been shown to be safe and efficacious both as a short- and long-term treatment for schizophrenia in both adults [13–20], and in children and adolescents [21,22]. Use of lurasidone is associated with a low risk for weight gain and metabolic abnormalities [23,24], which may be attributable, at least in part, to its lack of activity at 5HT_2C_ and histamine H_1_ receptors [25–27].

To date, no published studies have evaluated the efficacy and safety of lurasidone in the 13–25-year-age cohort of patients, a cohort that encompasses what is generally considered to be early onset of schizophrenia [28]. The current post hoc analysis attempts to address this issue. To have sufficient power to evaluate the efficacy and safety of lurasidone in this adolescent and young adult population, we have analyzed pooled results from six previously reported clinical trials.

## Methods

Individual data for patients aged 13–25 years were pooled from two sources: (a) five similarly designed, randomized, double-blind, placebo-controlled, 6-week studies of lurasidone in adult patients (aged 18–75 years) with schizophrenia [13–17]; and (b) one similarly designed, randomized, double-blind, placebo-controlled, 6-week study of lurasidone in adolescent patients (aged 13–17 years) with schizophrenia [21]. Patients included in these studies had a diagnosis of schizophrenia (based on Diagnostic and Statistical Manual of Mental Disorders, fourth edition [29] or fourth edition, text revision [30] with an acute exacerbation of psychotic symptoms as indicated by a Clinical Global Impression–Severity of Illness Scale (CGI-S) score of ≥4 (moderate or greater). Study entry required patients to meet the following minimum levels of symptom severity: a Positive and Negative Syndrome Scale (PANSS) total score of ≥80 (in three adult studies); a comparable score of ≥42 on the PANSS-derived Brief Psychiatric Rating Scale (in two adult studies) [31]; or a PANSS total score of ≥70 (in the adolescent study).

Key exclusion criteria were similar across all six pooled studies and included an acute or unstable medical condition; evidence of any other chronic disease of the central nervous system; alcohol or other drug abuse/dependence within the past 3–6 months; evidence of a severe, chronic movement disorder; or imminent risk of suicide (as judged by the study investigator).

Conduct of each study was consistent with the Good Clinical Practices guidelines of the International Conference on Harmonisation and with the ethical principles described in the Declaration of Helsinki. An independent Data and Safety Monitoring Board monitored each study. Prior to the conduct of any study procedures, written informed consent was obtained for young adults; and for adolescents, written informed consent was obtained from a parent or legal guardian, and assent was obtained from each adolescent patient.

Patients were randomized to receive placebo, or fixed-dose, once-daily, oral lurasidone (in the adult studies: 40, 80, 120, or 160 mg; in the adolescent study: 40 or 80 mg). Due to the small sample sizes in the 120- and 160-mg dosage groups in the adult studies, these two dosage groups were combined into a 120/160-mg lurasidone high-dosage group. Olanzapine [14] and quetiapine extended release (XR) [17] were used as active comparators in one study each; results for these active comparator arms were not included in the current analysis. Concomitant administration of lorazepam, temazepam, and zolpidem (for clinically significant anxiety/agitation, or insomnia), and anticholinergic agents or propranolol for movement disorders was permitted on an as-needed basis. In the adult studies, patients were hospitalized for the first 2–4 weeks of treatment and were then eligible for outpatient treatment, based on CGI-S scores and the judgment of the investigator. In the adolescent study, hospitalization was permitted for part or all of the study based on the judgment of the investigator.

Efficacy was assessed using the PANSS [32] and the CGI-S [33], which were administered at baseline, Day 3 or 4, Day 7, and weekly thereafter. Adverse events were recorded based on spontaneous report. Safety evaluations included body weight and laboratory tests (metabolic parameters and prolactin).

### Statistical analyses

Because individual patient-level data were available for all six studies, it was possible to perform a more powerful pooled analysis instead of a meta-analysis. This pooled analysis included all patients, aged 13–25, who were randomized and received at least one dose of study medication, and had PANSS efficacy assessments at baseline and at least one postbaseline time point. For each outcome measure, least-squares (LS) mean change from baseline was obtained from a mixed model for repeated measurement (MMRM) analysis. The model included fixed effects for study protocol, pooled site within study, visit as a categorical variable, baseline score, treatment score, and treatment by visit interaction. MMRM analysis of the PANSS included the PANSS total and subscale scores (positive, negative, and general psychopathology subscales). All significance tests were two-tailed with alpha = 0.05. Response rate was defined as ≥30% improvement at study endpoint. Significance testing was not adjusted for multiplicity. This is because these were post hoc analyses that are considered exploratory and we were not rigorously testing an *a priori* hypothesis. Number needed to treat (NNT) was calculated as the reciprocal of the difference in response rates for lurasidone versus placebo at LOCF-endpoint. Cohen’s *d* effect sizes for lurasidone were calculated as the between-treatment group difference in LS mean change score divided by the model estimate of the pooled standard deviation of the change scores. For individual adverse events, the number needed to harm (NNH) to obtain one additional instance of each event was calculated as the reciprocal of the difference in the incidence of each event between the lurasidone and placebo groups.

## Results

**Figure 1.**
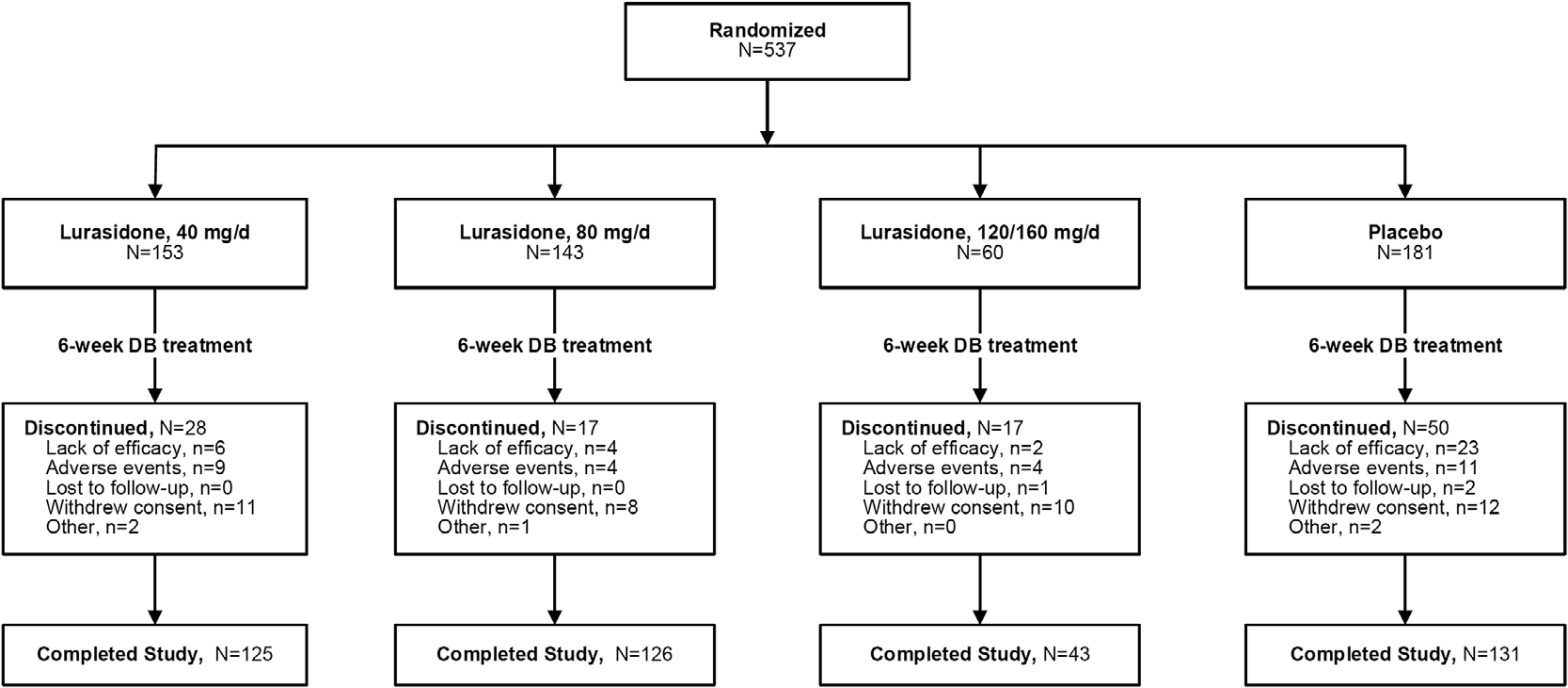
Patient disposition.

A total of 537 patients were included in this analysis (Figure 1). Baseline patient characteristics are summarized in Table 1. Only adult patients were randomized to the lurasidone 120/160-mg combined high-dosage groups. As a result, the mean age was somewhat higher and the mean PANSS scores were somewhat higher (due to a 10-point higher PANSS total score entry criterion in the adult studies) in the adult studies. Rates of premature study discontinuation were lower for lurasidone (all doses combined) compared with placebo (17.4 vs. 27.6%). There was a somewhat higher increase in overall discontinuation rate on lurasidone for the higher (120/160 mg) dosage group (28.3%) compared with the 40-mg group (18.3%) and the 80-mg group (11.9%; Figure 1). This difference was only partially attributable to discontinuations due to adverse events, which were comparable in the higher (120/160 mg) dosage group (6.7%) compared with the 40-mg group (5.9%) and the 80-mg group (2.8%).

**Table 1.** Baseline characteristics (safety population).

	Lurasidone			Placebo
	40 mg (*N* = 153)	80 mg (*N* = 143)	120/160 mg (*N* = 60)	(*N* = 181)
Male (%)	67.3	70.6	81.7	67.4
Age (years, mean [SD])	17.5 (3.5)	17.0 (3.3)	22.20 (2.2)	18.1 (3.9)
Race (%)				
White	60.8	62.9	41.7	54.1
Black	19.0	19.6	20.0	23.8
Asian	10.5	9.8	33.3	13.3
Other	9.8	7.7	5.0	8.8
Age at illness onset (mean [SD])	16.5 (2.1)	15.9 (2.5)	17.6 (2.8)	16.5 (2.6)
PANSS total score (mean [SD])	95.7 (12.2)	96.0 (11.6)	97.6 (13.3)	95.0 (11.8)
CGI-S (mean [SD])	5.0 (0.6)	4.9 (0.6)	5.0 (0.7)	4.9 (0.6)

*Abbreviations:* CGI-S, Clinical Global Impression–Severity Scale; PANSS, Positive and Negative Syndrome Scale.

### Efficacy

The LS mean changes from baseline to Week 6 in the PANSS total scores were significantly greater for all lurasidone dosage groups compared with placebo (*p* < 0.001). Effect sizes were moderate-to-large (40 mg = 0.53, 80 mg = 0.57, and 120/160 mg = 0.90; Table 2). A similar pattern of significance, and incremental dose-related increases in effect size, were also observed for the PANSS positive, negative, and general psychopathology subscales (Table 2). LS mean changes in the CGI-S scores were significantly greater than placebo for all lurasidone dosage groups, with *p* < 0.001 and effect sizes ranging from 0.51 to 0.96 (Table 2).

**Table 2.** Week 6 change from double-blind baseline in efficacy measures.

	LS mean (SE)	LSMD (95% CI)	Effect size	*p*-value
PANSS total				
Lurasidone 40 mg	−22.0 (1.6)	−9.4 (−13.5, −5.2)	0.53	<0.001
Lurasidone 80 mg	−22.7 (1.6)	−10.1 (−14.3, −5.9)	0.57	<0.001
Lurasidone 120/160 mg	−28.7 (2.6)	−16.1 (−22.0, −10.1)	0.90	<0.001
Placebo	−12.6 (1.5)			
PANSS positive subscale				
Lurasidone 40 mg	−7.5 (0.5)	−3.7 (−5.0, −2.4)	0.65	<0.001
Lurasidone 80 mg	−7.8 (0.5)	−4.0 (−5.3, −2.6)	0.70	<0.001
Lurasidone 120/160 mg	−9.8 (0.9)	−5.9 (−7.9, −4.0)	1.05	<0.001
Placebo	−3.8 (0.5)			
PANSS negative subscale				
Lurasidone 40 mg	−4.7 (0.5)	−2.1 (−3.3, −0.9)	0.42	<0.001
Lurasidone 80 mg	−4.7 (0.4)	−2.1 (−3.3, −0.9)	0.42	<0.001
Lurasidone 120/160 mg	−5.6 (0.8)	−3.0 (−4.8, −1.3)	0.60	<0.001
Placebo	−2.6 (0.4)			
PANSS general psychopathology subscale				
Lurasidone 40 mg	−9.6 (0.8)	−3.3 (−5.4, −1.2)	0.37	0.002
Lurasidone 80 mg	−10.1 (0.8)	−3.8 (−5.9, −1.7)	0.42	<0.001
Lurasidone 120/160 mg	−13.4 (1.4)	−7.1 (−10.2, −4.1)	0.80	<0.001
Placebo	−6.3 (0.8)			
CGI-S				
Lurasidone 40 mg	−1.13 (0.10)	−0.52 (−0.76, −0.27)	0.51	<0.001
Lurasidone 80 mg	−1.10 (0.10)	−0.49 (−0.73, −0.25)	0.48	<0.001
Lurasidone 120/160 mg	−1.60 (0.16)	−0.98 (−1.33, −0.64)	0.96	<0.001
Placebo	−0.62 (0.09)			
PANSS responders (≥30% improvement)	Responder rate (%; LOCF-endpoint)	NNT	Odds ratio (95% CI)	*p*-value
Lurasidone 40 mg	49.3	6	2.5 (1.6, 4.1)	<0.001
Lurasidone 80 mg	51.4	5	2.7 (1.7, 4.3)	<0.001
Lurasidone 120/160 mg	55.0	4	2.2 (1.1, 4.3)	0.025
Placebo	30.0			

*Notes:* Sample size: lurasidone 40 mg (*n* = 151), 80 mg (*n* = 145), 120/160 mg (*n* = 60); placebo (*n* = 180).

*Abbreviations:* CGI-S, Clinical Global Impression–Severity Scale; LSMD (95% CI), LS mean difference in Week 6 change scores for lurasidone versus placebo (with 95% confidence intervals); NNT, number needed to treat; PANSS, Positive and Negative Syndrome Scale.

**Figure 2.**
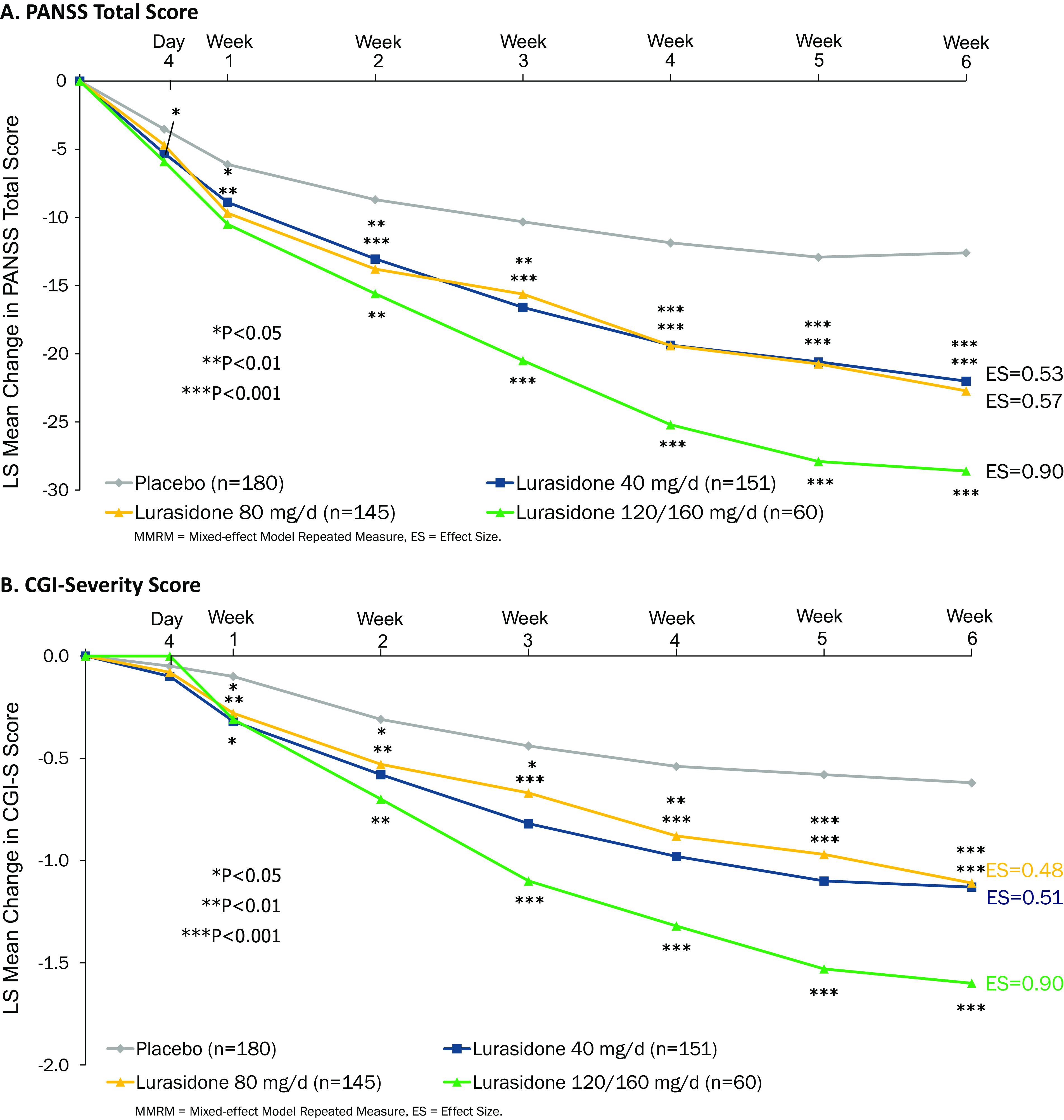
Change from double-blind baseline during 6 weeks of double-blind treatment with lurasidone doses (40–160 mg/d). (A) Positive and Negative Syndrome Scale total score. (B) Clinical Global Impression–Severity score.

Significant separation from placebo began to emerge by Week 1 on the PANSS total score and was consistently evident across all doses of lurasidone from Weeks 2 to 6 (Figure 2A). A similar early onset of treatment effects was observed for lurasidone on the CGI-S Scale (Figure 2B).

PANSS responder rates at LOCF-endpoint were significantly higher for all doses of lurasidone compared with placebo, with the NNT values of 6 for the 40-mg dose, 5 for the 80-mg dose, and 4 for the high-dose (120/160-mg) group (Table 2).

### Safety

The following adverse events occurred during treatment with lurasidone (all doses combined vs. placebo) at a rate of ≥2%: nausea (13.5 vs. 3.9%; NNH = 11), somnolence (12.1 vs. 5.0%; NNH = 15), akathisia (10.1 vs. 1.7%; NNH = 12), parkinsonism (4.2 vs. 0.6%; NNH = 28), dizziness (3.9 vs. 0.6%; NNH = 31), and dystonia (2.0 vs. 0.6%; NNH = 72). Table 3 summarizes adverse events separately for each lurasidone dosage group. Among patients in the high-dosage (120/160 mg) lurasidone group, a dose-related increase in the incidence of adverse events was evident for somnolence, akathisia, and a range of extrapyramidal symptoms.

**Table 3.** Adverse events (%; ≥5% on lurasidone).

	Lurasidone			Placebo
	40 mg (*N* = 153)	80 mg (*N* = 143)	120/160 mg (*N* = 60)	(*N* = 181)
Nausea	13.1	14.0	13.3	3.9
Somnolence	11.8	9.8	18.3	5.0
Akathisia	9.2	9.1	15.0	1.7
Parkinsonism	5.2	0.7	10.0	0.6
Dizziness	5.2	3.5	1.7	0.6
Dystonia	0	1.4	8.3	0.6
Drooling	0.7	0	5.0	0
Torticollis	0	0	6.7	0
At least one adverse event	68.0	62.2	70.0	63.0

Laboratory measures of cholesterol, triglycerides, and glucose showed no clinically meaningful differences between lurasidone and placebo (Table 4). Treatment with lurasidone was associated with a dose-related increase in median prolactin levels in female patients, most notably in the high-dosage (120/160 mg) lurasidone group (Table 4). In an individual dose analysis of the small sample of female patients, the median prolactin increase on lurasidone was +21.6 μg/L in the 120-mg dosage group (*n* = 5), and +14.7 μg/L in the 160-mg dosage group (*n* = 5).

**Table 4.** Change from Double-blind baseline in laboratory values and weight/BMI (LOCF-endpoint).

	Lurasidone			Placebo
	40 mg (*N* = 153)	80 mg (*N* = 143)	120/160 mg (*N* = 60)	(*N* = 181)
Metabolic (mg/dL; median change)				
Total cholesterol	−4.00	−1.50	+0.00	−7.00
Triglycerides	−2.00	+6.00	−10.00	−2.00
Glucose	0.00	+1.00	−2.00	+1.00
Prolactin (μg/L; median change)				
Female	+0.40	+5.95	+21.35	0.00
Male	+0.27	+1.10	+1.00	−0.80
Weight (kg)	0	0	0	0
LOCF-endpoint change (mean [SD])	+0.41 (2.16)	+0.69 (2.13)	+1.05 (2.03)	+0.12 (2.61)
≥7% increase in weight (%)	4.1	2.2	6.0	4.7
≥7% decrease in weight (%)	2.7	0.7	0	2.4

The proportion of patients experiencing a clinically meaningful (≥7%) dose-related increase in weight was approximately similar for all three lurasidone dosage groups compared to placebo (for the high-dosage (120/160 mg) lurasidone group vs. placebo, the NNH was >50**;** Table 4).

## Discussion

In this multiregional, pooled six-study analysis of adolescents and young adults (aged 13–25 years) with an acute episode of schizophrenia, short-term treatment with lurasidone demonstrated statistically significant efficacy compared to placebo across the dose range of 40–160 mg/d. Efficacy results appeared to be robust, as indicated by the consistently significant reduction in symptom severity observed for all doses of lurasidone on all efficacy measures. Improvement in schizophrenia symptom severity was clinically meaningful as suggested by the magnitude of the effect sizes, which ranged from 0.53 to 0.90 on the primary efficacy measure, the PANSS total score. As is typical with first and second generation antipsychotic agents (with the exception of clozapine) [34], the magnitude of improvement on lurasidone was greater on the PANSS positive subscale compared with the PANSS negative subscale (effect sizes: 0.65–1.05 vs. 0.42–0.60). Onset of improvement on lurasidone was noted as early as Week 1 and was consistently observed from Weeks 2 to 6 across the 40–160-mg/d dose range. Early onset of improvement was also observed on the global CGI-S measure.

There is a relative lack of published data evaluating the efficacy of atypical antipsychotics as a class in the adolescent and young adult population reported here. The current results are comparable to results reported in a comprehensive network meta-analysis of randomized controlled trials in adolescents with schizophrenia [35], which comprises the younger half of the current analysis sample. Out of a total of 28 antipsychotic agents included, the meta-analysis found clozapine to be the most efficacious antipsychotic (vs. placebo), followed by olanzapine, risperidone, and lurasidone.

Lurasidone was notably safe and well-tolerated in this adolescent and young adult patient population. For lurasidone doses combined, the NNH relative to placebo was ≥10 for all individual adverse events. A step-wise, dose-related increase in akathisia and extrapyramidal adverse events was noted when comparing treatment with the lower (40/80 mg) versus the higher (120/160 mg) doses of lurasidone. The same dose-related increase was evident in the incidence of somnolence. However, it should be noted that the current data are from fixed-dose studies in which dose adjustments for tolerability were not permitted. Nonetheless, the double-digit NNH values observed in this pooled analysis, and the low (5.2%) discontinuation rate due to adverse events, suggest that lurasidone is well tolerated in this young population. This finding is consistent with meta-analyses that have found lurasidone to be one of the best tolerated among all available antipsychotic medication in both adolescents [35,36] and in adults [34].

Short-term treatment with lurasidone was found to have a favorable safety profile in this adolescent and young adult population, with minimal effects on metabolic parameters and prolactin. As expected, there was a small dose-related effect on prolactin noted in females. These findings are consistent with results from a network meta-analysis of pediatric patients with schizophrenia [35] which found lurasidone to have less effect on prolactin when compared with risperidone, olanzapine, haloperidol, and paliperidone. However, more safety data are needed regarding the effects of higher doses of lurasidone in young females, since the current sample size (*n* = 10) was very small in the combined 120/160-mg dosage group.

Treatment effects on weight were also small and not different from placebo to a clinically meaningful degree. The minimal effects of short-term treatment with lurasidone on weight, prolactin, and metabolic parameters are consistent with previous comparative meta-analyses that have identified lurasidone as having minimal effects on these safety parameters [35–37].

A few study limitations should be noted. Most importantly, the current results represent a post hoc pooled analysis, with no adjustment made for multiplicity, and thus the results should be considered exploratory. In addition, the sample size for the two combined high-dosage groups (120 and 160 mg) were relatively small; therefore, the efficacy and tolerability results should be viewed as tentative.

In adolescents and young adults with a diagnosis of schizophrenia, choice of drug often represents a potential commitment to an especially long course of treatment with the chosen agent. While efficacy is commonly considered the primary factor that determines choice of drug, the tolerability and safety profile is perhaps as important as efficacy in making this treatment decision. Poor tolerability has been associated with reduced adherence to long-term therapy, and an increase in hospitalization rates [37]. Similarly, the diagnosis of schizophrenia is associated with high rates of obesity, diabetes, metabolic syndrome, and a 20-year shorter life span than individuals without a diagnosis of schizophrenia, primarily due to increased cardiovascular mortality [38]. Over the past decade, there has been increasing evidence suggesting that widely prescribed antipsychotic agents may iatrogenically contribute to the adverse weight and metabolic profile occurring in patients with chronic schizophrenia [35,38]. Bearing in mind the considerations summarized above, the efficacy, tolerability, and safety results reported in this pooled analysis suggest that lurasidone has a favorable benefit-risk profile that makes it suitable to consider among first-line treatments of schizophrenia in adolescent and young adults.

## Data Availability

The data that support the findings of this study are available upon reasonable request from Dr. Robert Goldman at Sunovion Pharmaceuticals Inc.
